# Family planning among people living with HIV in post-conflict Northern Uganda: A mixed methods study

**DOI:** 10.1186/1752-1505-5-18

**Published:** 2011-09-20

**Authors:** Barbara Nattabi, Jianghong Li, Sandra C Thompson, Christopher G Orach, Jaya Earnest

**Affiliations:** 1 Centre for International Health, Faculty of Health Sciences, Curtin University, Perth, Western Australia, Australia; 2 Combined Universities Centre for Rural Health, University of Western Australia, Geraldton, Western Australia, Australia; 3 Centre for Population Health Research and Curtin Health Innovation Research Institute, Faculty of Health Sciences, Curtin University, Perth, Western Australia, Australia; 4 Telethon Institute for Child Health Research, Perth, Western Australia; Australia; 5 Department of Community Health and Behavioral Sciences, Makerere University School of Public Health, Kampala, Uganda

**Keywords:** HIV/AIDS, contraception, mixed methods, Northern Uganda

## Abstract

**Background:**

Northern Uganda experienced severe civil conflict for over 20 years and is also a region of high HIV prevalence. This study examined knowledge of, access to, and factors associated with use of family planning services among people living with HIV (PLHIV) in this region.

**Methods:**

Between February and May 2009, a total of 476 HIV clinic attendees from three health facilities in Gulu, Northern Uganda, were interviewed using a structured questionnaire. Semi-structured interviews were conducted with another 26 participants. Factors associated with use of family planning methods were examined using logistic regression methods, while qualitative data was analyzed within a social-ecological framework using thematic analysis.

**Results:**

There was a high level of knowledge about family planning methods among the PLHIV surveyed (96%). However, there were a significantly higher proportion of males (52%) than females (25%) who reported using contraception. Factors significantly associated with the use of contraception were having ever gone to school [adjusted odds ratio (AOR) = 4.32, 95% confidence interval (CI): 1.33-14.07; p = .015], discussion of family planning with a health worker (AOR = 2.08, 95% CI: 1.01-4.27; p = .046), or with one's spouse (AOR = 5.13, 95% CI: 2.35-11.16; p = .000), not attending the Catholic-run clinic (AOR = 3.67, 95% CI: 1.79-7.54; p = .000), and spouses' non-desire for children (AOR = 2.19, 95% CI: 1.10-4.36; p = .025). Qualitative data revealed six major factors influencing contraception use among PLHIV in Gulu including personal and structural barriers to contraceptive use, perceptions of family planning, decision making, covert use of family planning methods and targeting of women for family planning services.

**Conclusions:**

Multilevel, context-specific health interventions including an integration of family planning services into HIV clinics could help overcome some of the individual and structural barriers to accessing family planning services among PLHIV in Gulu. The integration also has the potential to reduce HIV incidence in this post-conflict region.

## Background

Between 1987 and 2007, Northern Uganda was affected by civil conflict resulting in a complex humanitarian emergency, characterized by a displacement of over 1.5 million people from their homes into overcrowded internally displaced persons (IDP) camps. The region experienced an increase in transmission of infectious diseases and increased mortality rates [1]. In 2006 Northern Uganda had the highest infant mortality rates (106 deaths per 1,000 live births) and under-five mortality (177 deaths per 1,000 live births) in all of Uganda, with even higher rates in the IDP camps at 123 and 200, respectively [2]. During the insurgency, disruptions to the health care system and social infrastructure, and migration of skilled health workers to more stable parts of the country led to limited availability of, and access to, quality health services among the IDPs [1].

Consequent to the insurgency, Gulu District had the highest percentage of its population (58.1%) in the lowest quintile of wealth in Uganda, and only 0.9% of females and 3.0% of males had completed secondary education [2]. Northern Uganda also had the lowest use of contraceptives by currently married women aged 15-49 years: only 10.9% of women were using family planning methods in 2006 [2]. The total unmet need for family planning in the Northern region was 46% among currently married women (compared with 41% nationally), with 29.5% of these women unable to access family planning services to help space births and 16.5% unable to limit their family size. Overall, only 19.1% of total demand for family planning was being met in Northern Uganda, the lowest percent in the whole country and the total fertility rate was 7.5 children, one of the highest rates in the country [2].

Despite being a largely rural area, in 2004, the prevalence of HIV for the North Central Uganda region reached 8.2% (9.0% for women and 7.1% for men), one of the highest in Uganda, and in contrast to a national average of 6.4% and other predominantly rural areas such as the West Nile region (2.3%) [3]. The displacement of populations, food insecurity leading to transactional and survival sex, where sex was exchanged for basic survival with an element of exploitation by older, and wealthier men, and rape by combatants were considered to be the key drivers of the high prevalence of HIV in post-conflict Northern Uganda [4].

However, despite the poor health and social indicators in Northern Uganda [1,2], there is limited information about PLHIV in the region, especially around individual, social, cultural and structural impediments to health care due to the protracted conflict, which limits evidence-based allocation of resources. Other quantitative studies have documented factors associated with contraception use among PLHIV in Uganda [5-7] but the circumstances in Northern Uganda warrant a detailed exploration. Underpinned conceptually by the Social Ecological Framework which proposes that an individual's behavior is influenced by several factors at a multitude of levels [8,9], this mixed-methods study aimed to determine the knowledge of, access to and, factors associated with use of family planning methods among PLHIV in Gulu District, Northern Uganda.

## Methods

### Setting

Gulu District is situated in the Acholi-sub region of Northern Uganda and has a population of 581,740 people [10]. According to the 2002 Uganda census, a quarter (25%) of the population was living in Gulu town, with the rest either in IDP camps or in the rural areas [11]. Gulu town, the economic capital of the northern region, is 332 kilometers north of the capital city, Kampala.

### Recruitment of respondents

A mixed-methods design constituting a survey and semi-structured interviews was selected for this study. Between February and May 2009, 476 PLHIV were recruited to take part in the study. These respondents attended three HIV clinics within Gulu municipality area: St. Mary's Hospital, Lacor, Gulu National Referral Hospital (GNRH) and The AIDS Support Organization (TASO) clinic. The sample size was calculated on the premise that 50% of the sample would desire to have children (the key outcome of the overall larger study), with an acceptable sampling error of 5% and at 95% level of confidence. The selection criteria for respondents in this study were HIV-infected women and men aged 15-49 years, attending outpatient HIV clinics in Gulu District, and consenting to participate in the study, regardless of length of time attending the clinic or highly active antiretroviral therapy (HAART) history. Pre-determined quotas by clinic, age and sex were used to ensure that a sufficient number of respondents for both sexes and relevant age groups were recruited. Thus equal proportions (14.3%) of respondents were recruited in each age group i.e. 15-19, 20-24, 25-29, 30-34, 35-39, 40-44, and 45-49 year groups. Seven trained interviewers approached consecutive clients attending these three clinics and asked them to participate in the study and recruitment continued until these quotas were filled.

### Data collection procedures

A 121-item questionnaire was administered to each respondent to collect socio-demographic information, sexual and reproductive history, family planning knowledge and use, fertility desires and intentions and experiences of stigma. The questions on women's and men's fertility desires and contraceptive use were adopted from the 2006 Uganda Demographic and Health Survey (UDHS) [2]. For the purpose of this study, contraception use was defined as the use of any modern or traditional method to prevent a pregnancy [2]. Modern methods included female and male sterilization, the oral contraceptive pill, intrauterine device, injectables, implants, male and female condoms, lactational amenorrhoea and emergency contraception. Traditional methods included periodic abstinence and withdrawal.

To collect information about family planning knowledge, the respondents were asked to name ways or methods by which a couple could delay or avoid pregnancy. If a respondent failed to mention a particular method spontaneously, the interviewer described the method and asked whether the respondent had heard of it, and if they had ever used the method. This form of prompting was used in case the respondent knew the method by another name or knew the method but not its name. The respondents were asked if they were currently using any method to prevent a future pregnancy. For this study, use of contraception by the spouse was also considered use by the respondent: for example, if the husband of the female respondent was using condoms, she was considered to be using condoms as a contraception method.

The respondents were asked where they obtained contraception and sources of information on family planning methods, methods they preferred, reasons for not using contraception, and whether health workers at the facility had ever discussed family planning with them. The respondents in long term stable relationships (married or de facto) and those who were separated, divorced or widowed were also asked if they had discussed family planning methods with their spouses in the past. They were also asked about the status (alive or dead) and sex of their biological children. The female respondents were asked if they were currently pregnant. All respondents were asked whether they desired to have children in the future.

The respondents were also asked about HIV transmission routes and antiretroviral therapy. They were also asked about the length of time since HIV diagnosis, the length of time attending the HIV clinic, if they were on highly active antiretroviral therapy (HAART) and, if so, the length of time on HAART. The respondents in long term relationships and those who were separated, divorced or widowed were also asked about their spouses' HIV status and if they had disclosed their own HIV status to their spouse. Complete knowledge about prevention of mother-to-child transmission (PMTCT) was defined as being able to correctly name the three routes of HIV transmission from mother to child i.e. during pregnancy, delivery and while breastfeeding.

For the qualitative arm of the study, three interviewers explored the experiences of family planning and service provision with 26 participants, using a semi-structured guide. The selection criteria for these participants were being HIV-infected, aged 15-49 years, living in Gulu District and consenting to participate in the study. These semi-structured interviews were held in the privacy of the participants' homes, out of hearing range of other family members and neighbours to ensure confidentiality. The interviews lasted between 1-2 hours. All the interviews were conducted in Luo, audiorecorded, then transcribed and translated into English.

The first author also interviewed United Nations Population Fund (UNFPA) staff members, managers of Marie Stopes International, Uganda and Reproductive Health Uganda and Family Health International, and officials from the Ministry of Health, Uganda in order to determine the availability and coverage of HIV and family planning services in Gulu. These officials were also asked about the amount and sources of funding for family planning services for the general population, whether there were specific family planning programs for PLHIV and the level and type of family planning training that health workers had received.

The study received ethical approval from the Curtin University Human Research Ethics Committee, the Makerere University School of Public Health Institutional Review Board, and the Uganda National Council for Science and Technology (UNCST). In order to ensure that respondents were able to give informed consent, the interviewers read out a prepared translated information sheet where the respondents were informed about the objectives, procedures and implications of the study. Respondents were informed that they were free to withdraw at any stage in the study and provided either written or thumb-printed consent.

### Analyses

Quantitative data were analyzed using SPSS Statistics Version 19 for Windows (SPSS Inc, Chicago, Illinois, USA). Socio-demographic characteristics and the reproductive and HIV history of the respondents were summarized using proportions for categorical variables and medians with interquartile ranges for continuous variables. Separate analyses were conducted for males and females to determine the magnitude of differences in knowledge of contraception, current family planning use and preferred family planning methods. Bivariate analysis was conducted to determine the association between current use of family planning and the independent variables. Factors significantly associated at the p < .10 level in bivariate analysis with current use of family planning were evaluated in multivariate logistic analysis. A sub-analysis was conducted to determine the factors independently associated with current use of barrier and hormonal methods of contraception, because they serve different purposes and require different actors for their use. The former methods of contraception also function to prevent HIV/STI transmission and mainly require male participation and cooperation while the latter are used by females. The strengths of associations are presented as odds ratios (OR) or adjusted odds ratios (AOR) with 95% confidence intervals (CI).

Qualitative data were managed using Nvivo8 software (QSR International Pty Ltd). Interview transcripts were systematically read and reread to ensure familiarity with the content, and initially coded using an open coding method [12]. A coding framework was developed to identify dominant themes and subthemes related to family planning experiences. Some of the themes were adopted from the literature, while others emerged from the data. The cases and quotes that illustrate the themes best [13] are presented in this paper.

## Results

### Quantitative results

#### Sample characteristics

Four hundred and seventy six respondents (238 males and 238 females), were recruited into this arm of the study. Ninety eight respondents (20.6%) were from Gulu National Referral Hospital (GNRH), 168 (35.3%) from St. Mary's Hospital, Lacor, and 210 (44.1%) from The AIDS Support Organization (TASO) clinic (Table 1). Eighty two percent of respondents had ever attended school, but 45.9% (179/390) had less than 7 years of primary education, and only 6.4% (25/390) had attended university or other tertiary institutions. Seventy two percent were of the Roman Catholic religion. Fifty percent of respondents were in a long term stable relationship (married or de facto), with 28.4% of these respondents in polygamous relationships; 46.9% were peasant farmers, and 48.3% were living in urban areas (towns/trading centres).

**Table 1:** Sociodemographic characteristics, reproductive and HIV history of PLHIV in Gulu District, Uganda, February-May 2009 (n = 476)

Characteristic	Number	Percent
**Sex**		
Male	238	50.0
Female	238	50.0
**Clinic attended**		
GNRH	98	20.6
Lacor	168	35.3
TASO	210	44.1
**Education**		
Never attended school	85	17.9
Some primary education	179	37.6
Completed primary education	84	17.6
Some secondary education	87	18.2
Completed secondary	16	3.3
Tertiary education	25	5.3
Missing	1	0.2
**Religion**		
Roman Catholic	340	71.5
Other	131	27.5
Missing	5	1.0
**Relationship status**		
Never married	76	15.9
Married/De facto	236	49.6
Separated/Divorced/Widowed	164	34.5
**Polygamy (if married/de facto)**		
Monogamous	169	71.6
Polygamous	67	28.4
**Occupation**		
Peasant farmers	222	46.9
Professionals	24	5.1
Others	230	48.0
**Residence**		
Town/Trading centre	230	48.3
Village	204	42.9
IDP camp	41	8.6
Other	1	0.2
**Reproductive history**		
Respondents who have ever had children	397	83.4
Respondents who had ever lost a child	137	34.9
Currently pregnant (females only)	18	7.6
**Respondents on HAART **^a^	236	49.8
**Time on HAART (months) **^b^		
Less than 24 months	112	47.7
24 months and more	123	52.3
**Spouse's HIV status **^c ^		
Positive	213	53.3
Negative	49	12.1
Not applicable/unknown/missing	138	34.6
**Disclosure of HIV status to spouse **^c ^		
Yes	268	66.9
No	61	15.3
Unknown/missing	71	17.8
**Complete PMTCT knowledge**	319	67.0

GNRH, Gulu National Referral Hospital; HAART, Highly Active Antiretroviral Therapy; IDP, Internally displaced people; TASO, The AIDS Support Organization; ^a ^data for 2 respondents missing; ^b ^data for one person on HAART missing; ^c ^single respondents excluded

Eighty-three percent of the respondents had ever had children and 34.9% (137/392) had also lost a child. Eighteen female respondents (7.6%) were pregnant at the time of the study. The median number of children born to the respondents was 3 (interquartile range 1-5). Fifty percent of the respondents were on HAART, with 52.3% of them having been on HAART for 24 months or longer. Of the respondents in long term stable relationships or those who had been separated, divorced and widowed, 53.5% had an HIV positive spouse. Eighty one per cent (268/329) had disclosed their HIV status to their spouse. Sixty-seven per cent of the respondents knew all the three routes of HIV transmission from mother to child i.e. during pregnancy, delivery and while breastfeeding.

#### Knowledge and use of family planning

The majority of respondents (96%) knew at least one method of family planning (Table 2). Fifty nine percent had discussed family planning with a health worker while 62.6% of those in long term relationships or separated/divorced/widowed had ever discussed family planning with their spouse. Though 70% of all respondents had used a family planning method in the past, only 38% were currently using any method. Twenty seven percent were currently using a barrier method of contraception. While there was no difference in knowledge of, and past use of family planning methods by sex, there were statistically significant differences in the proportion of male and female respondents who had discussed family planning with health workers and spouses, and those currently using family planning methods. Significantly more women (66%) had discussed family planning with health workers than men (56%), but conversely, significantly fewer women (54%) had ever discussed family planning with their spouse in comparison to the male respondents (72%). About half of the male respondents (52%) reported that they were currently using a method of family planning, compared to only 25% of the female respondents.

**Table 2:** Family planning knowledge, discussion and use among PLHIV in Gulu District, Uganda, February-May 2009 (n = 476):

Variable	All n = 476	Males n = 238	Females n = 238	p value *
	**n (%)**	**n (%)**	**n (%)**	

Have knowledge of at least one family planning method	457 (96)	230 (97)	227 (96)	0.482
Have ever discussed family planning with health workers^a^	281 (59)	129 (56)	152 (66)	**0.037**
Have ever discussed family planning with their spouse^b, c^	224 (63)	122 (72)	102 (54)	**0.000**
Have past history of using any family planning method	330 (70)	165 (69)	165 (70)	1.000
Currently using any form of family planning^d^	181 (38)	121 (52)	60 (25)	**0.000**
Currently using a barrier method of family planning	126 (27)	100 (42)	26 (11)	**0.000**

* p value calculated using Pearson's chi square; ^a ^missing data for 15 respondents; ^b ^single respondents excluded; ^c ^missing data for 42 respondents; ^d ^missing data for 8 respondents

The male condom was the most commonly known method (99.4%), followed by the pill (88.3%) and injectables (87.5%). Male condoms were also the most commonly used form of contraception (69.2%), followed by the injectables (19.4%), then periodic abstinence (10.9%). Among the respondents who were currently using contraception methods, eighty-two percent of males compared to 41.3% of females were using the male condom. However, only 17% were using dual methods, that is, a male condom and another method at the same time. In Uganda, the condom is generally promoted as a means to prevent HIV transmission rather than as a family planning method [5]. When the male condom was excluded from the analysis, only 18% (88/476) of the respondents were using a method generally considered as a means of preventing pregnancy. The majority of the clients preferred to use condoms (30.3%), followed by injectables (28.7%) and the pill (17.1%). Most of the respondents had heard about family planning on the radio (89.4%) and other sources of information included newspapers (25.5%), posters (25.4%), TV (8.7%) and video (11.7%).

Forty-three percent (184/430) of the respondents desired to have more children, significantly more males than females (54.2% vs. 31.7% respectively; Pearson's chi square = 35.248, d.f. = 1, p = .000). Of the 246 respondents who said they did not desire to have any more children, 59.3% (146) were not using any method to prevent further pregnancies: 34% of the 97 men and 76% of the 148 women who reported they did not want any more children, were not using any form of contraception. There was no difference in whether respondents had discussed family planning with health workers by clinic attended (Pearson's chi square = .030, d.f. = 1, p = .863).

Bivariate analysis (Table 3) showed that current family planning use was significantly associated (at the p < .05 level) with being male, being married or in a de facto relationship, having ever gone to school, having at least one child, not having had a death of a child, having discussed family planning with a health worker and spouse, attending TASO or Gulu National Referral clinics, having adequate knowledge about PMTCT, and spouse's lack of desire for children. In multivariate analysis (Table 3), having ever gone to school [adjusted odds ratio (AOR) = 4.32, 95% confidence interval (CI): 1.33-14.07; p = .015], discussion of family planning with a health worker (AOR = 2.08, 95% CI: 1.01-4.27; p = .046), or with one's spouse (AOR = 5.13, 95% CI: 2.35-11.16; p = .000), not attending the Catholic-based clinic (AOR = 3.67, 95% CI: 1.79-7.54; p = .000) and spouse's non-desire for children (AOR = 2.19, 95% CI: 1.10-4.36; p = .025) remained significantly associated with the current use of contraception.

**Table 3:** Factors associated with current family planning use among PLHIV in Gulu District, Uganda, February-May 2009

Variable	Total n	Currently using a family planning method n (%)	OR (95% CI)	p value	AOR (95% CI)	p value
Age group ^a^						
15-29 years	199	68 (34.2%)	1.00			
30-49 years	269	113 (42.0%)	1.39 (0.95-2.04)	0.085		
Sex ^a^						
Female	236	60 (25.4%)	1.00		1.00	
Male	232	121 (52.2%)	3.19 (2.17-4.72)	**0.000**	1.90 (0.91-3.97)	**0.085**
Marital status ^a^						
Single/divorced/widowed	232	48 (20.7%)	1.00		1.00	
Married/de facto	236	133 (56.4%)	4.90 (3.26-7.37)	**0.000**	2.19 (0.98-4.88)	**0.055**
Type of marriage (if married or de facto) ^b^						
Polygamous	66	37 (56.1%)	1.00			
Monogamous	169	95 (56.2%)	1.01 (0.57-1.79)	0.983		
Residence ^c^						
Rural	241	90 (37.3%)	1.00			
Urban	226	91 (40.3%)	1.13 (0.78-1.64)	0.517		
						
Education ^c^						
No	85	17 (20.0%)	1.00		1.00	
Yes	382	164 (42.9%)	3.03 (1.69-5.26)	**0.000**	4.32 (1.33-14.07)	**0.015**
Number of children ^c^						
0 children	73	20 (27.4%)	1.00			
1 child and more	394	161 (40.9%)	1.83 (1.05-3.18)	**0.030**		
History of death of child ^d^						
Yes	135	42 (31.1%)	1.00			
No	225	117 (45.9%)	1.88 (1.21-2.91)	**0.005**		
Discussion of family planning with health workers ^e^						
No	179	43 (24.0%)	1.00		1.00	
Yes	278	137 (49.3%)	3.07 (2.03-4.66)	**0.000**	2.08 (1.01-4.27)	**0.046**
Discussion of family planning with spouse ^f, g^						
Never	134	20 (14.9%)	1.00		1.00	
At least once	224	131 (58.5%)	8.00 (4.65-13.89)	**0.000**	5.13 (2.35-11.16)	**0.000**
HIV Clinic attended ^a^						
Lacor (faith-based hospital)	164	50 (30.5%)	1.00		1.00	
Others (GNRH and TASO)	304	131 (43.1%)	1.73 (1.16-2.58)	**0.008**	3.67 (1.79-7.54)	**0.000**
On HAART ^c^						
Yes	231	87 (37.7%)	1.00			
No	236	93 (39.4%)	1.08 (0.74-1.56)	0.699		
HIV status of spouse ^f, h^						
Negative	49	26 (53.1%)	1.00			
Positive	212	114 (53.8%)	1.03 (0.55-1.92)	0.928		
Disclosure of HIV status to spouse ^f, i^						
Yes	267	128 (47.9%)	1.00			
No	60	27 (41.7%)	0.78 (0.44-1.37)	0.379		
Months since HIV diagnosis ^j^						
Less than 24 months	215	81 (37.7%)	1.00			
24 months or more	247	99 (40.1%)	1.11 (0.76-1.61)	0.597		
Months on HAART ^k^						
Less than 24 months	111	37 (33.3%)	1.00			
24 months or more	119	49 (41.2%)	1.40 (0.82-2.39)	0.219		
Months attending HIV clinic ^l^						
Less than 24 months	258	97 (37.6%)	1.00			
24 months or more	208	81 (38.9%)	1.06 (0.73-1.54)	0.766		
Complete PMTCT knowledge ^a^						
Yes	315	133 (42.2%)	1.00			
No	153	48 (31.4%)	0.63 (0.42-0.94)	**0.024**		
Desire for children ^m^						
Yes	184	72 (39.1%)	1.00			
No	246	100 (40.7%)	1.06 (0.72-1.57)	0.750		
Religion						
Other	128	51 (39.8%)	1			
Catholic	335	129 (38.5%)	0.95 (0.62-1.43)	0.792		
Spouses' desire for children (if married or de facto) ^n^						
Yes	87	43 (49.4%)	1		1	
No	83	59 (71.1%)	2.51 (1.34-4.74)	**0.004**	2.19 (1.10-4.36)	**0.025**
Any HIV-infected children (among those with children) °						
Yes	75	38 (50.7%)	1			
No	223	97 (43.5%)	0.75 (0.44-1.266)	0.281		

AOR, adjusted odds ratio; CI, confidence interval; GNRH, Gulu National Referral Hospital; HAART, highly active antiretroviral therapy; OR, odds ratio; TASO, The AIDS Support Organization; ^a ^data for 8 respondents missing; ^b ^data for one person missing; ^c ^data for 9 respondents missing; ^d ^data for 7 respondents missing; ^e ^data for 19 respondents missing; ^f ^single respondents excluded; ^g ^data for 42 respondents missing; ^h ^data from 139 respondents missing; ^i ^data from 73 respondents missing; ^j ^data from 14 respondents missing; ^k ^data from 6 respondents missing; ^l ^data from 10 respondents missing; ^m ^data from 46 respondents missing; ^n ^data from 66 respondents missing; ^o ^data from 2 respondents missing

On further multivariate analysis of the association between the independent variables and the current use of barrier methods and hormonal methods, the following remained significant: male sex (AOR = 7.29, 95% CI: 3.73-14.29), being in a stable relationship (AOR = 4.46, 95% CI: 2.04-9.80), discussion of family planning with one's spouse (AOR = 9.06, CI: 3.98-20.61), and not attending the Catholic-based clinic (AOR = 4.75, 95% CI: 2.44-9.28) were significantly associated with use of barrier methods. Being in a stable relationship (AOR = 2.30, 95% CI: 1.09-4.85), and discussion of family planning with a health worker (AOR = 5.62, 95% CI: 2.03-15.62) were significantly associated with use of hormonal contraception.

### Qualitative results

Six key themes around factors influencing contraception use among PLHIV were identified from the analysis of semi-structured interviews with clients and staff in the various organizations: personal barriers to using contraception, perceptions of family planning methods, decision making, covert use of contraception, targeting females for family planning services, and structural barriers to using contraception (summary in Table 4).

**Table 4:** Main themes from the semi-structured interviews with PLHIV in Gulu, Northern Uganda

**Personal barriers to using contraception**	Bad experiences with using some methods, fear of side effects, health concerns, and reduced sensation.
	Spousal opposition to family planning methods
	Religious affiliation
**Perceptions of family planning methods**	Positive perceptions
	Negative perceptions (among clients and health workers): • To condoms • To male vasectomy
**Decision making**	Male dominated
**Covert use of family planning methods**	Women surreptitiously receive injectables or implants at family planning clinics
	Clients keep the records at the health centre
**Targeting of females for family planning services**	Program managers mainly targeted females
	Men reluctant to do vasectomy but send spouses for sterilization
	Client perception that family planning was women's business
**Structural barriers to using contraception**	Lack of health workers trained in family planning provision and counselling
	Very few doctors in the region as a result of the civil conflict
	Only two family planning clinics based in Gulu town serving the whole population
	Male and female sterilization services delivered by Kampala-based medical staff
	Family planning services did not specifically target PLHIV
	No specific family planning programs for PLHIV in HIV clinics
	Lack of referral systems and lack of collaboration between health facilities

#### Personal barriers to using contraception

All the participants had heard about family planning methods but the majority were not currently using any method, consistent with the quantitative findings. Reasons for the low level of use included bad experiences with using some methods, fear of side effects, and health concerns. Some participants reported that for these reasons they would never use contraception again. It became clear that, after one bad experience, individuals often were reluctant to use alternative methods or took some time to do so. One female participant said:

*"Yes. The injectable one, but it mistreated badly and I stopped it. I will never try again"*. 

Another participant said:

*"After I started using the drug I got side effect then I went back to the hospital and they told me to stop using it; I was using Depo injectable and they told me it was the one causing the side effect. And I have not used family planning method since then, but I want to go and start using another method if possible"*.

In some cases, there was spousal opposition to family planning methods. A female participant who was unable to use the contraceptive pill because of severe side effects was asked if her husband uses condoms and she responded:

*"No, he doesn't allow to use them"*.

Some opposition was due to male concerns about experiencing reduced sensation while using the condom. One 40-year-old male participant said:

*"Condom, I don't know how to use condom and you don't enjoy your sweet when it is wrapped"*. 

For others, religious affiliation was an inhibiting factor for using contraception. One male participant said:

*"He *[the health worker] *advised me to use condom and other methods. And I told him I cannot use condom because I am a Catholic, and you can't control birth"*.

#### Perceptions of family planning methods

Some clients had perceived family planning positively and they believed that family planning services helped families in a number of ways:

*"I think their service is important because it helps a lot by reducing the burden on parents"*. 

Opportunities to obtain advice on contraception were seen as important for both women and their children, as described by a female participant:

*"Yes, I am advocating for the service to continue, because it helps people in spacing their children, therefore it helps in the proper growth of children and gives mother some resting period from one child to another"*.

Other participants perceived some methods as potentially harmful, a perception sometimes based upon misunderstanding or misinformation. One male participant said:

*"There are some bad cases of condom because if you don't use it well you may lose one's life.... it can get stuck in the vagina... there are some coils used by women that can damage condoms"*.

UNFPA officials reported that male vasectomy was unpopular in this region. Some women believed that male sterilization would affect their husband's sexual performance, and some health workers were reluctant to recommend permanent methods to their clients:

*"The health worker told me that child birth should be spaced but you should not be given a drug which will stop you from having children forever. You should use family planning so that you space your children and they will not be weak and sickly"*.

#### Decision-making

From the interviews with both men and women, it was apparent that males dominated in the decision making around fertility issues. While some female participants reported that they had discussions with their spouses about fertility and contraceptive use, ultimately the husband made the final decision. One female participant, who was interviewed after her husband, refuted his claim of using condoms to prevent more pregnancies:

*"We always discuss this with him, but when he is drunk he reneges on what we have agreed together. ... That why I told you that we can decide on not having any more children, but when he drinks he changes his mind and start demanding for another baby, but his other family members don't like the idea"*.

A woman's reliance upon her husband to provide condoms even when she didn't want more children was another problem identified:

*"I have never gone for one though I hear about, but we do use condom all the time and it is my husband who bring it. When he has forgotten, we just meet without it"*.

This comment reflects passivity and a lack of control or assertiveness over their own fertility that was found in several female participants interviewed.

#### Covert use of family planning methods

Some women preferred to use injectable forms of contraception because it allowed them to prevent further pregnancies without their husband's knowledge. The family planning service providers indicated that many women preferred to keep the records at the health centre so that their use of the services could be kept discreet. Attempts to use family planning covertly could result in severe consequences, as described by a family planning manager: A client's husband who detected implants she had surreptitiously received at a family planning clinic threatened to cut off her arm because she had unilaterally made a major family decision, which he regarded was his to make. This attitude was further affirmed by a key informant:

*'Once women are paid for at marriage, they do not have any say in the home. They are not expected to make any major decisions'*.

Due to concerns arising from these attitudes, some women preferred contraceptive methods such as Depo provera where their husband would not need to know, and for which he would not have to give consent.

#### Targeting of females for family planning services

Program managers affirmed a low level of male involvement in family planning in general and admitted that their programs mainly targeted females, a feature which irked some men in the community. Several men told health workers that their programs would fail because they were targeting the 'wrong' people. However, there was a perception by some men and women that family planning was women's business. As one male participant said:

*"They should provide women with information on the radio programme, and they organize meetings at the sub-counties where women are informed about family planning...not only wait when the women go to the hospital, but the health worker should come to the community and inform the women"*.

Family planning managers confirmed that while some men would send their women for sterilization, they were reluctant to undergo sterilization themselves. However, the covert use of family planning indicates that some female participants made unilateral decisions and accessed family planning without their spouse's knowledge and permission.

#### Structural barriers to using contraception

Based on the interviews with the family planning service providers, few health workers in Gulu were trained in family planning provision and counselling due to the inability of organizations to provide training services to health workers during the period of insurgency. According to the UNFPA officials, there were very few doctors in the region as a result of the civil conflict, and yet these were the cadre of health workers they preferred to train in surgical contraceptive procedures. There were only two family planning clinics based in Gulu town, run by Marie Stopes International Uganda (MSIU) and Reproductive Health Uganda (RHU), serving the whole population in Gulu and surrounding districts. Clients were mainly self-referred.

Most of the hormonal and barrier methods, except for the female condom, were available at these two facilities. However, male and female sterilization services were not provided directly at these clinics and were only available as part of mobile surgical clinics when medical staff could be deployed from the capital city over 300 kilometres away. These occasional outreach mobile services were unable to meet the needs of the PLHIV who wished to limit their family sizes. Overall, the family planning services provided to the general population did not specifically target PLHIV. Within the three HIV clinics, only TASO clinic provided counselling services and provided clients with free condoms. Thus, there was no systematic integration of reproductive health services in the HIV clinics, and there was lack of referral systems and collaboration between health facilities for family planning services.

## Discussion

This study has documented the level of knowledge of, and factors associated with family planning use among a PLHIV population in the resource-poor, post-conflict region of Northern Uganda. We found a very low level of current family planning use despite a high level of knowledge about contraceptive methods. Factors associated with using family planning methods in this PLHIV population included having ever gone to school, discussion of family planning with a health worker or with one's spouse, not attending the Catholic-based clinic and spouse's non-desire for children. Discussion with a spouse have also been found to be associated with use of hormonal contraceptives in Rakai, Uganda [7]. Religion also has an impact on the uptake of contraception [14], through its influence at both the individual level and the institutional level, where faith-based health facilities may not directly provide family planning services to clients, thus limiting the access by PLHIV to these services.

Fear of side effects, reduction in pleasure, misinformation, negative perceptions, and gender-inequality have also been identified in other studies as barriers to adopting family planning [14-16]. As found in other studies [5,17], male sterilization was not used: Strong aversion to vasectomy has been linked to fear of male impotence in some societies [18,19], and/or reluctance to terminate males' reproductive career [14]. Our study also showed low use of dual methods of contraception among PLHIV. Use of a barrier method in combination with other contraceptives maximizes contraceptive efficiency and reduces the risk of HIV transmission to sexual partners [17].

PLHIV in our study who did not desire to have more children were often unable to access the family planning services they needed. The lack of association between desire to have children with use of family planning methods in this PLHIV population could be explained by the structural barriers that exist in Northern Uganda as a consequence of the long period of conflict in the region, which led to the outmigration of skilled health workers, the limited number of existing family planning clinics, and lack of provision of family planning services within the HIV clinics. The generally low level of contraception use may be explained by the high level of desire for children in this population which may arise from esteem associated with large families [14], and low levels of female autonomy and literacy.

The strong desire to have children in this population may be further influenced by the prolonged civil conflict and high levels of infant and child mortality. Families, including couples living with HIV, which have lost their children during the conflict to either disease or violence, may have a strong desire to have more children. In societies with low literacy, endemic poverty, high child mortality and lack of social welfare and security programs, children are considered as a form of insurance to provide support in old age. Furthermore, having children in Uganda increases a person's social status [20] and this also applies to couples living with HIV.

Family planning programs and health workers mainly target women for family planning, but it is apparent that this approach did not result in discussion with their spouses or uptake of family planning services. Whether or not condoms were used was very much determined by the male spouse, particularly when the relationship was unstable. Our study showed that proportionally more females than males had discussed family planning with health workers. However, females generally reported not having discussed family planning with their spouse, whereas males reported high levels of spousal discussion on family planning, suggesting the focus of such discussions may have a different perspective for males and females. Fewer women than men reported using any method. Considering that men are the reproductive decision-makers in most traditional Ugandan homes [14], it is essential that reproductive health services also target men, educate them, and involve them in reproductive educational programs.

The ecological framework, as applied in this study, views the use of contraception among PLHIV as the outcome of interaction of factors at several levels: individual, interpersonal, and structural. At the individual level factors include demographic factors such as education status, sex, as well as personal attitudes and experiences of contraception. At interpersonal level, discussions and interactions with health workers, and spouses impact on the use of contraception. At the structural level, limited provision of family planning services in the general population and lack of integration of these services within HIV clinics inhibited the use of contraception among PLHIV. The usefulness of this framework is that it allows development of multi-level strategies to address the issue. Understanding the interdependency of factors at each level allows a holistic, and more effective approach to improving access while taking into account broader public health considerations.

Integration of family planning services with HIV services utilising a multi-level approach to improve the uptake is urgently needed in this region. Family planning programs should cater to PLHIV who wish to limit their family size, and also to those who wish to continue to have more children with a goal of achieving better health outcomes for the PLHIV through birth spacing and use of effective and safe contraception. Such integration has potential not only to improve reproductive health outcomes [21-24], but to ultimately reduce paediatric HIV infections [25], and hence reduce the amount of antiretroviral therapy needed. This is particularly important in countries such as Uganda where MTCT at 18% of new infections is a major route of HIV transmission [4].

Several levels of integration are possible. Family planning education should be provided within the HIV clinics and integrated into routinely provided general education programs with information on the effectiveness, safety, and possible side effects of all contraceptive methods. Doctors, nurses, and community workers attached to the HIV clinics could be trained in family planning counselling for PLHIV, and contraceptives could be provided free. Health workers can facilitate discussions of family planning with couples, either at health facilities or in the communities, and by doing so they can assist women in broaching the subject to their spouses and hence improve family planning use. HIV clinics have regular and prolonged contact with HIV-infected clients, and are ideally placed to meet their reproductive health needs over time [26]. While there has been some success in integration at PMTCT clinics [27], this is a temporary contact with HIV-infected clients that lasts only for the duration of pregnancy. Women generally do not return for post-natal family planning counselling [27], and PMTCT clinics target only women, whereas HIV clinics can target both men and women.

Family planning services can also be provided at the facility level, where clients are referred to separate clinics within the same health facility. It is also possible to have an active district-wide referral and follow-up service so that clients are appropriately referred to facilities that provide the service. Faith-based health facilities that may not directly provide family planning counselling and services can become part of a referral network. Although no difference was seen in this study between respondents' family planning discussions with health workers by the clinic they attended, actual use of family planning methods were significantly different, suggesting a need for active referral systems. Surgical contraceptive services should be readily available, sustainably funded, and provided by locally-trained doctors who could also deliver services at more remote clinics on a rotational basis. Nursing staff, in collaboration with community village health workers, could counsel and prepare clients for operations that are available on a regular schedule. The suggested measures could be coordinated and implemented by the local district health departments in collaboration with health facilities, local community organizations, government agencies, and UN partners. Though possible constraints include lack of time due to large client numbers and commodity shortages, local government health departments could determine funding sources, training requirements and implementation strategies.

This is the first study on family planning use among a PLHIV population in a conflict/post conflict region and it adds to the literature on family planning use among male and younger PLHIV. The majority of previous studies have examined family planning use among women only. Information from females alone is insufficient, particularly in the context of a patrilineal and male-dominant society. By documenting use of family planning among males, their access to and perceptions of its use, a clearer and more holistic picture of why their spouses may or may not be using contraception is revealed. The sampling approach also ensured that the outcomes of interest (family planning use) could be assessed on adequate numbers of males and females in the different age groups as well as allowing statistical comparisons across sex and age groups. Additionally the combination of quantitative and qualitative methods has provided important information about the use of family planning methods. The quantitative findings provided us with information on the level of knowledge of and use of family planning among this PLHIV population and reveal the variables independently associated with the use of family planning. The qualitative data highlight gender inequality and limited access to and poor quality of available contraceptives as important contributing factors for the low use of family planning among PLHIV. The qualitative methods also allowed for exploration of additional concepts not captured in the survey questionnaire, such as covert use of contraceptives by women and targeting of women by family planning programs.

Limitations of this study include the cross-sectional design and, hence, causality cannot be determined. The non-random sampling and recruitment at the health facilities also result in a bias towards clients who are able to access health facilities, who are more urban-based or wealthier than those who had no access. The younger respondents aged 15-19 years and male respondents may have been more prone to positive health-seeking behaviours than their counterparts in the general population. Social desirability bias may have occurred when respondents were interviewed: PLHIV may feel that they have to indicate that they are using condoms to prevent further spread of the infection, especially if condom use has been previously promoted by health workers. While the ratio of males to females in this sample is similar to that in the general HIV population in Northern Uganda, caution needs to be exercised in generalizing findings to the general HIV population. Nevertheless, the findings provide important information about factors that are associated with use or non-use of family planning methods and, despite the unique complexities of this post-conflict region, may have implications for HIV populations elsewhere.

Future studies could consider comparison of HIV-infected with non-infected clients to determine the impact of HIV on access to family planning and its use. Research on the general PLHIV population is needed to measure unmet needs for family planning services among PLHIV. Interviewing couples separately to ascertain reported condom use is recommended for future research.

## Conclusions

This study has documented a high level of knowledge but low use of family planning methods among a PLHIV population in post-conflict Northern Uganda, particularly among female PLHIV. Various individual and structural challenges prevent PLHIV from accessing the services they require. Integration of family planning services and education into HIV clinics could help ensure that these services become readily accessible to PLHIV and this would be a significant progress towards HIV prevention and reduction of HIV incidence in this post-conflict region.

## Competing interests

The authors declare that they have no competing interests.

## Authors' contributions

BN designed the study, collected and analysed the data, and prepared the initial draft. JL, SCT, CGO and JE assisted with the design of the study, and contributed to the interpretation of the results, reviewed the various drafts and assisted with the writing. All authors have read and approved the final manuscript for submission to a peer reviewed journal.

## Authors' information

BN is a medical doctor and public health practitioner from Uganda. She has worked extensively with people living with HIV in Northern Uganda managing one of the major HIV clinics in the region. She has a well-developed understanding of the socio-cultural determinants and structural factors that impact on health seeking behaviour and the necessity for appropriate research methods to elucidate health problems in the region. CGO is a medical doctor and public health physician in Uganda and has vast experience with working with similar populations. He is currently a Senior Lecturer and Head of Department of Community Health and Behavioural Sciences at the School of Public Health, Makerere University. SCT is a medical doctor, public health physician and Winthrop Professor of Rural Health at the University of Western Australia and currently Director of the Combined Universities Centre for Rural Health. Her research interest is in vulnerable populations especially the Indigenous population in Australia. JL is a social epidemiologist and her research focuses on social, economic and cultural determinants of health. She is currently a Senior Research Fellow at the Centre for Population Health Research at Curtin University and Associate Professor at Telethon Institute for Child Health Research. JE is a sociologist and educator whose research focuses on vulnerable populations and post conflict nations. She is currently Associate Professor at the Centre for International Health and Director of Graduate Studies in the Faculty of Health Sciences at Curtin University.
